# Supplementary material for: Amagmatic hydrothermal systems on Mars from radiogenic heat
Source: Nat Commun. 2021 Mar 19;12:1754. doi: 10.1038/s41467-021-21762-8 (PMC7979869; doi:10.1038/s41467-021-21762-8)
Supplement: Supplementary file 1 — Supplementary Information [file 41467_2021_21762_MOESM1_ESM.pdf]

Supplementary Online Materials

Amagmatic hydrothermal systems on Mars from radiogenic heat

Lujendra Ojha[1], Suniti Karunatillake[2], Saman Karimi[3], Jacob Buffo[4]

[1]Department of Earth and Planetary Sciences. Rutgers, The State University of New Jersey. Piscataway, NJ, USA.

[2]Department of Geology and Geophysics, Louisiana State University.

[3]Department of Earth and Planetary Sciences. Johns Hopkins University. Baltimore, MD, USA.

[4]Thayer School of Engineering, Dartmouth College, Hanover, NH, USA.

Corresponding author: Lujendra Ojha (Luju.ojha@rutgers.edu)

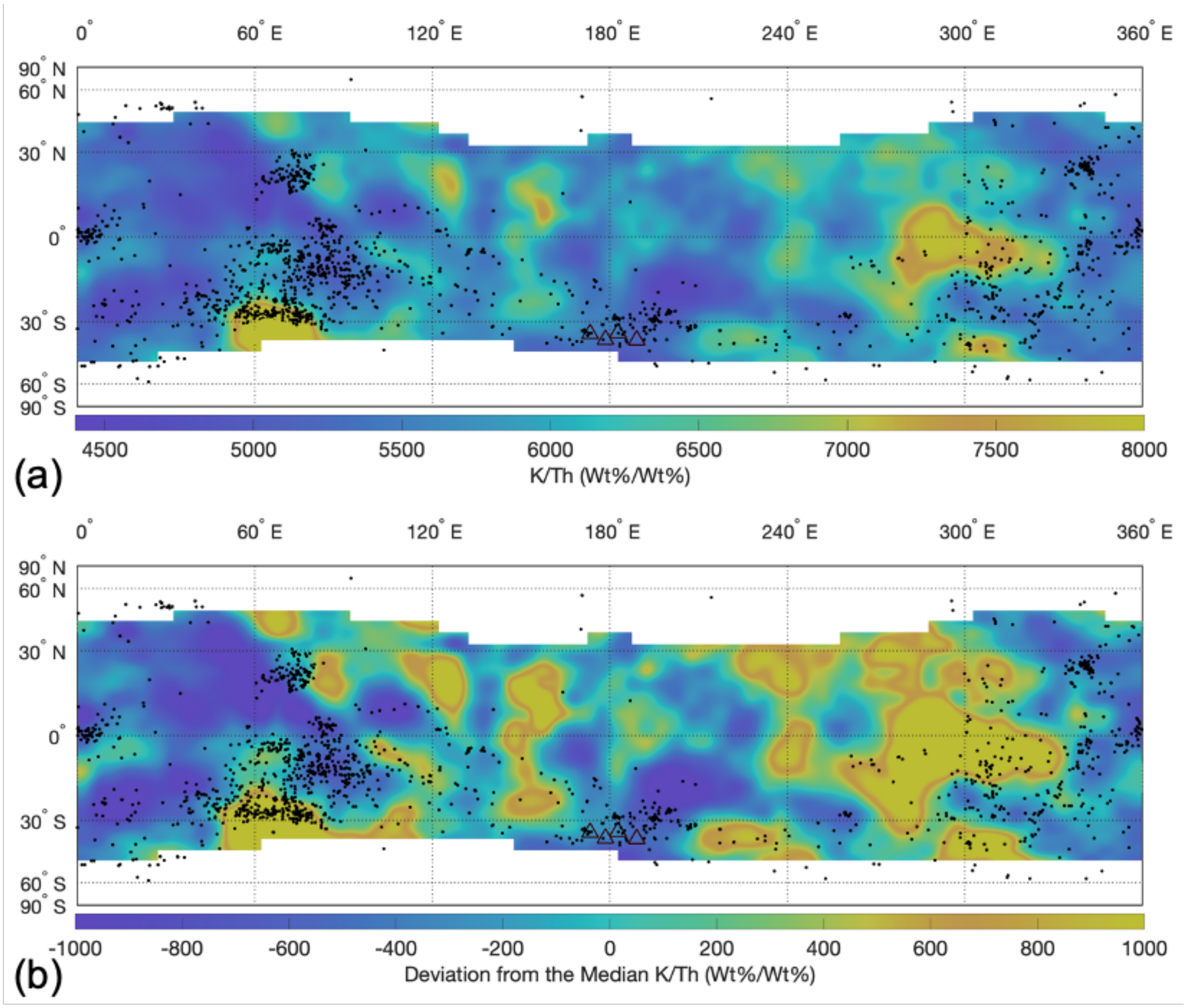

Supplementary Figure 1. *Map of the distribution of K/Th ratio on Mars. (a) Map showing the ratio of K/Th on Mars overlaid by the known distribution of phyllosilicates (black dots). The black triangles show the location of Eridania basins. (b) Map showing the deviation from the median value of K/Th on Mars. Regions with blue color have lower K/Th than the median K/Th value for Mars. Regions with yellow color have higher K/Th values than the median K/Th value.*

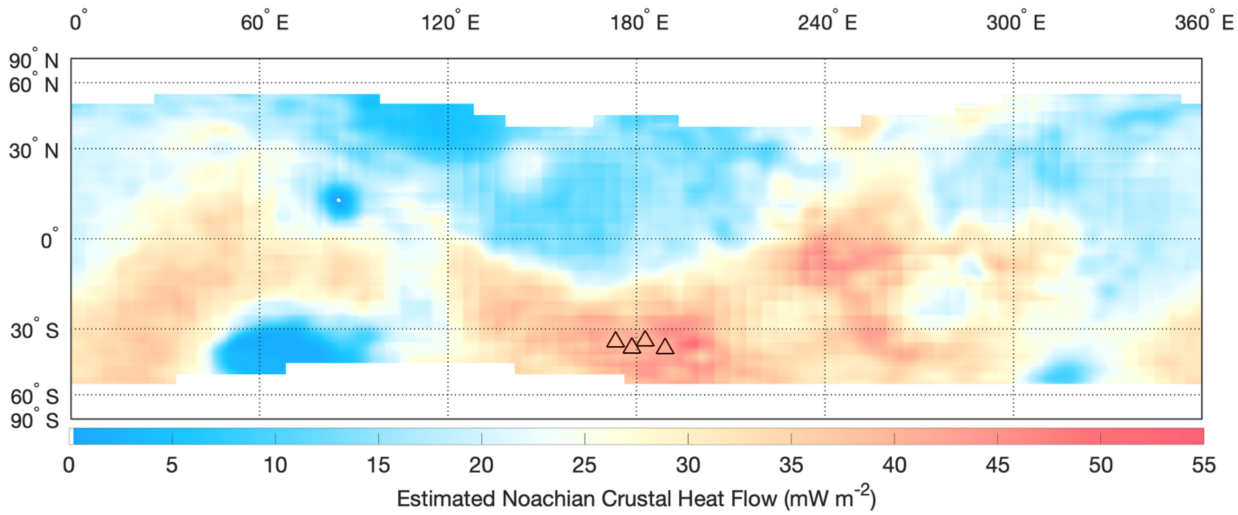

Supplementary Figure 2. *Surface heat flow estimates for Noachian Mars.* ***(A)*** *Crustal heat flow based on GRS-derived K and Th along with cosmochemically-equivalent U (Fig. 2) and crustal thickness map of Mars during the Noachian. An H mask has been applied to exclude areas with high H content. The black triangles show the location of Eridania basins.*

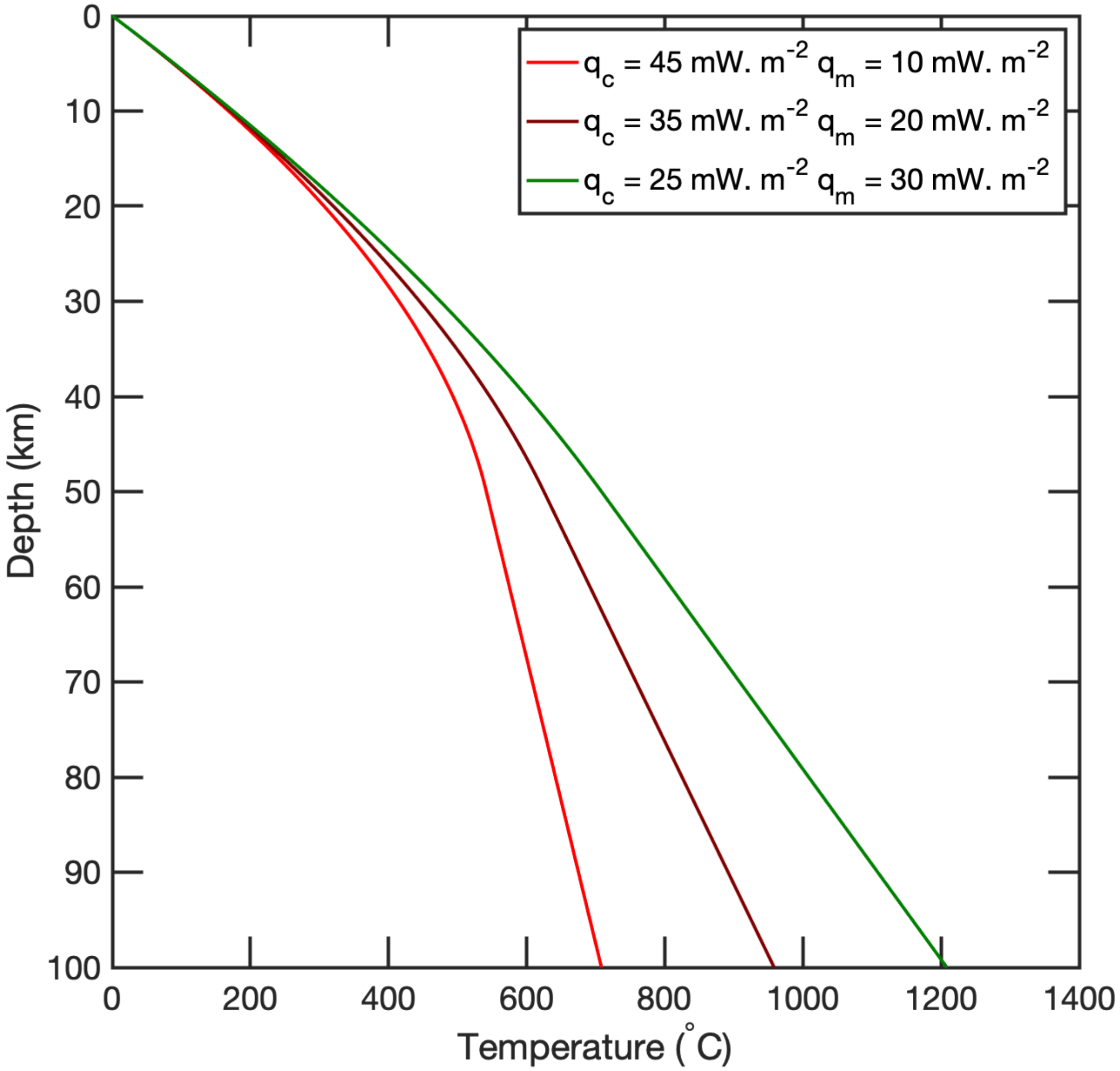

Supplementary Figure 3. *The temperature profile of the Martian lithosphere assuming q_s of 55 mW showing the effect of crustal fractionation of heat producing elements. The colored lines show the steady-state temperature profile for various values of crustal (q_c) and mantle (q_m) heat flow. The models assume that the surface heat flow in Eridania does not exceed 55 mW m^{-2}.*

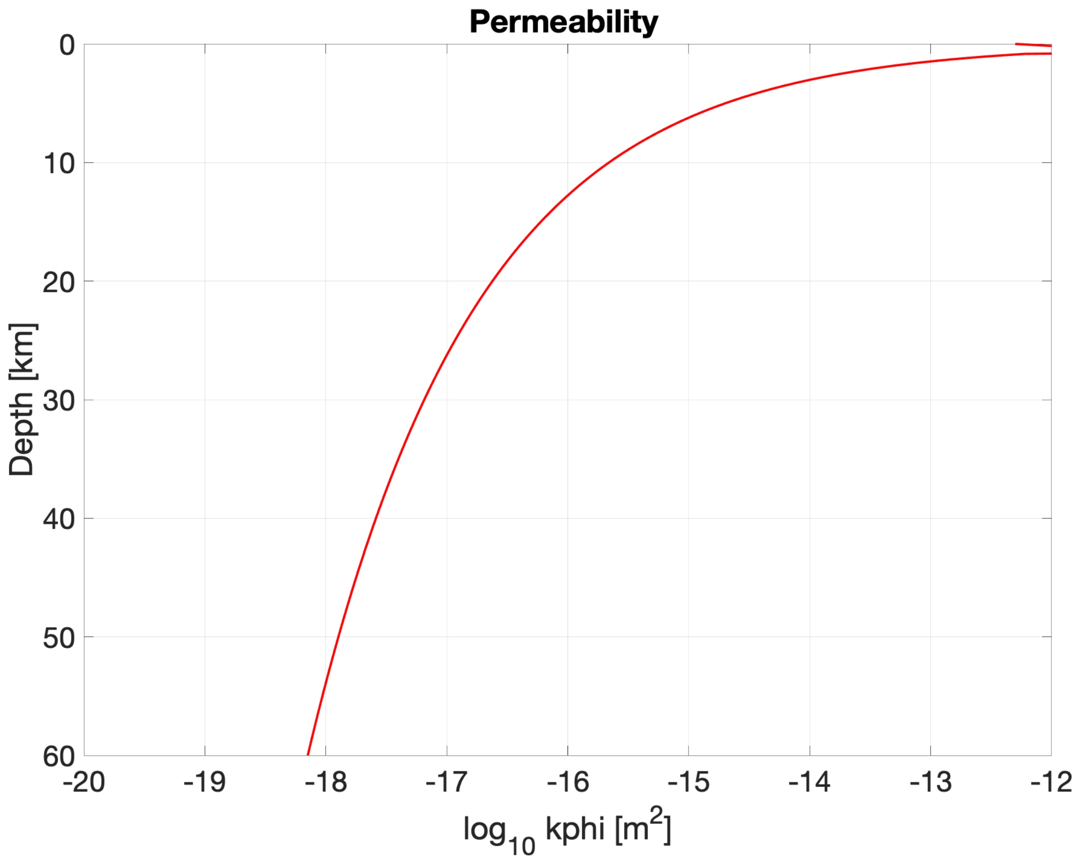

Supplementary Figure 4. *Permeability profile for Mars as a function of depth. The permeability profile was scaled for Mars using observed empirical permeability profile for the Oregon Cascades region on Earth.*

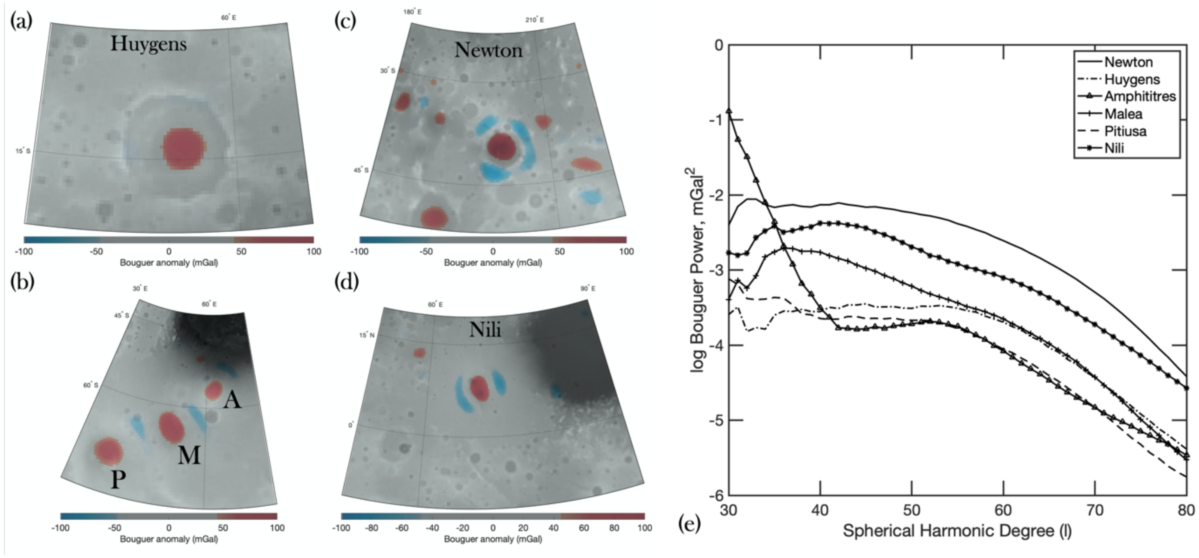

Supplementary Figure 5. *Filtered Bouguer gravity anomaly map of various regions on Mars and their corresponding localized power spectra.* ***(a)*** *Huygens is a large Noachian aged impact crater. Figure* ***(b)*** *shows three Noachian aged volcanoes Pitiusa, Malea, and Amphititres.* ***(c)*** *Bouguer gravity anomaly associated with Newton crater. Figure* ***(d)*** *shows a Noachian aged volcano, Nili Patera.* ***(e)*** *The localized power spectrum for the regions shown in (a) – (d).*

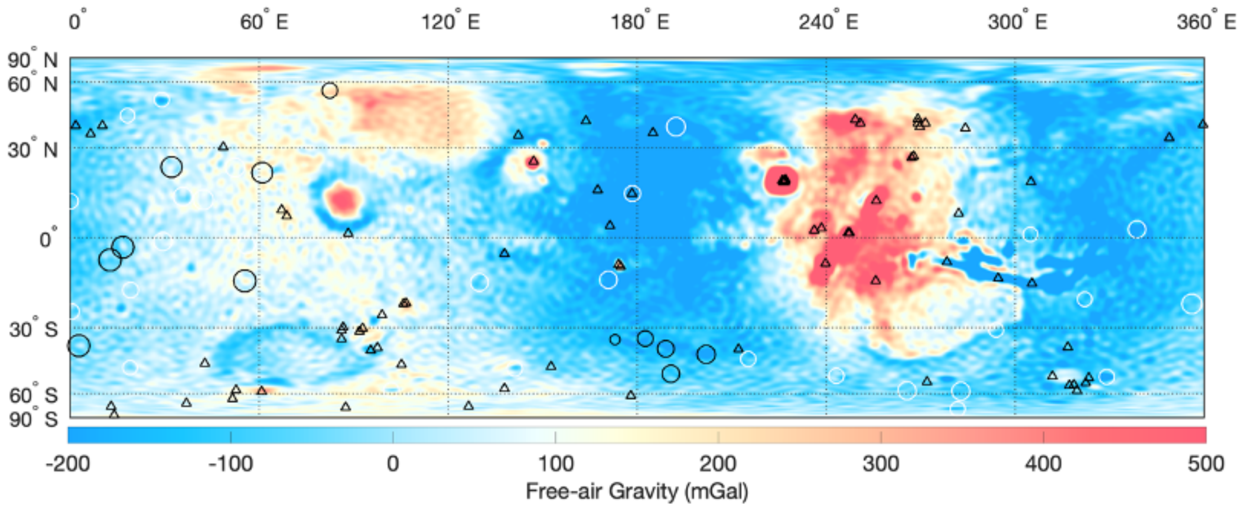

Supplementary Figure 6. *Free-air gravity anomaly map of Mars. The circles show impact craters on Mars with a diameter between 200 and 500 km. Impact craters with associated Bouguer anomaly are shown in black while white circles show impact craters with no prominent Bouguer anomaly (see Fig. 5). The triangles show all identified volcanic structures on Mars.*

Table S1. *Table listing craters on Mars with a diameter between 200 to 500 km and their name, location, depth, diameter, depth/diameter ratio, and gravity information.*

Site Number	Crater Name	Lon (E)	Lat (N)	Depth (km)	Diameter (km)	d/D	Bouguer Gravity?
1	~	42.287	12.284	0.79	391.18	2.02E-03	0
2	~	356.036	-21.637	0.42	382.56	1.10E-03	0
3	~	52.429	22.949	0.52	353.44	1.47E-03	0
4	~	35.849	13.187	1.74	344.88	5.05E-03	0
5	~	192.391	38.034	0.29	339.46	8.54E-04	0
6	~	29.458	-1.246	0.13	325.07	4.00E-04	0
7	~	265.688	-58.19	0.85	318.55	2.67E-03	0
8	~	282.769	-58.419	0.44	315.66	1.39E-03	0
9	Herschel	129.89	-14.397	1.32	303.82	4.34E-03	0
10	de vaucouleurs	170.964	-13.598	0.91	298.22	3.05E-03	0
11	~	338.768	2.735	1.97	291.51	6.76E-03	0
12	Schroeter	55.943	-2.297	1.08	290.06	3.72E-03	0
13	Dolfus	0.386	-24.19	0.54	252.26	2.14E-03	0
14	~	178.493	14.144	1.78	251.59	7.08E-03	0
15	Flaugergues	19.182	-16.897	1.19	245.68	4.84E-03	0
16	~	243.168	-50.056	0.48	229.63	2.09E-03	0
17	Kepler	141.106	-46.789	1.99	227.75	8.74E-03	0
18	~	0.208	11.766	0.31	224.37	1.38E-03	0
19	~	215.361	-42.353	0.61	223.91	2.72E-03	0
20	Galle	329.064	-50.664	3.64	223.12	1.63E-02	0
21	Secchi	101.907	-57.881	1.95	221.66	8.80E-03	0
22	Lyot	29.157	50.233	3.14	216.53	1.45E-02	0
23	Schmidt	281.794	-72.298	1.61	213.76	7.53E-03	0
24	~	304.834	1.133	1.09	211.91	5.14E-03	0
25	~	293.988	-30.752	1.69	211.25	8.00E-03	0
26	~	322.172	-19.955	0.71	208.47	3.41E-03	0
27	Kaiser	19.109	-46.131	1.62	206.5	7.85E-03	0
28	~	18.179	42.864	0.81	203.77	3.98E-03	0
29	Eridania - II	182.602	-34.145	1.6	240.52	6.65E-03	+
30	SW-Schiaparelli	12.732	-7.023	0.82	482.45	1.70E-03	+
31	Huygens	55.536	-13.793	1.44	476.79	3.02E-03	+
32	Schiaparelli	16.823	-3.072	1.14	468.29	2.43E-03	+
33	Greeley	2.813	-36.851	0.95	463.53	2.05E-03	+
34	Cassini	32.126	23.104	1.73	425.4	4.07E-03	+
35	Antoniadi	60.969	21.154	1.12	420.53	2.66E-03	+
36	Newton	201.896	-40.337	3.47	322.89	1.07E-02	+
37	Eridania	189.185	-38.09	1.87	271.88	6.88E-03	+

38	~	82.441	54.905	0.2	233.18	8.58E-04	+
39	Copernicus	190.8	-49.2	1	294	3.40E-03	+
40	Eridania-III	173	-34.5	~	~		+
